# Factors Affecting the Outcome of Medical Treatment in Cats with Obstructive Ureteral Stones Treated with Tamsulosin: 70 Cases (2018–2022)

**DOI:** 10.3390/vetsci9100568

**Published:** 2022-10-16

**Authors:** Hyung-Kyu Chae, Hyun Jeong Hong, Se Yoon Lee, Jung-Hoon Park, Woo Joo Choi, Seungkuk Oh, Seoyeoun Ji, Yeon-Jung Hong

**Affiliations:** 1 Department of Veterinary Internal Medicine, Western Referral Animal Medical Center, Seoul 04101, Korea; 2 Laboratory of Veterinary Internal Medicine, College of Veterinary Medicine, Seoul National University, Seoul 08826, Korea; 3 Department of Statistics, Texas A&M University, College Station, TX 77843, USA; 4 Department of Veterinary Radiology, Western Referral Animal Medical Center, Seoul 04101, Korea; 5 Department of Veterinary Surgery, Western Referral Animal Medical Center, Seoul 04101, Korea

**Keywords:** α-antagonist, cat, tamsulosin, ureteral obstruction, ureterolithiasis

## Abstract

**Simple Summary:**

Other than surgical approaches the treatment options for feline ureterolithiasis are limited compared to those of human medicine. After various studies on ureteral muscle relaxation drugs, tamsulosin has been used as a treatment for distal ureteral stones in human medicine. However, the available clinical veterinary data on the efficacy of drugs that relax the ureter are limited. Thus, this study aimed to investigate the factors affecting the outcome of tamsulosin treatment for feline ureterolithiasis. With tamsulosin treatment, the ureteric stone passage was confirmed in 22 out of 70 cats, with a success rate of 31.43%. Negative factors with statistical significance for stone passage in this study were high baseline creatinine levels, female sex, proximal location of stones, and large diameter stones. The results of this study suggest that tamsulosin can be considered for the treatment of ureterolithiasis in cats with small distal ureteral stones. In addition, this study serves as an important reference for determining whether medical treatment of feline ureteral obstruction using tamsulosin can be attempted.

**Abstract:**

The incidence of diseases associated with feline ureteral obstruction is increasing; however, non-surgical treatment options are limited. This study evaluated the outcome of medical treatment in cats with obstructive ureteral stones treated with tamsulosin and identified potential factors predicting spontaneous stone passage. We retrospectively reviewed 70 client-owned cats treated at the Western Referral Animal Medical Center, Seoul, Korea, from 2018 to 2022. All the cats had obstructive ureterolithiasis and were treated using tamsulosin. The baseline characteristics of the cats, stone diameter and location, and stone passage outcomes were analyzed. Stone passage occurred in 22 cats; the remaining 48 cats showed no change in stone locations. Sex, creatinine, and diameter and location of stones were potential risk factors associated with successful stone passage, but age, weight, and side of the stone were not. No serious adverse events related to tamsulosin treatment were observed. This is the first study to identify the risk factors predictive of the spontaneous stone passage of cats with obstructive ureterolithiasis after tamsulosin treatment. Tamsulosin could be an alternative treatment for ureteral obstruction in male cats with smaller distal ureteral stones and low baseline serum creatinine levels. These findings could help develop guidelines for treating feline ureterolithiasis.

## 1. Introduction

The incidence of diseases related to feline ureteral obstruction has been increasing recently [1]. Depending on the degree of obstruction, cats may be asymptomatic or exhibit symptoms related to severe kidney damage [2]. In cases of complete obstruction, prompt and appropriate treatment is required, since kidney function decreases proportionally with the increase in the duration of obstruction [3]. The causes of ureteral obstruction include ureterolithiasis, ureteral strictures, infections, ureteral ectopia, and neoplasia [4]. According to the results of several studies, ureterolithiasis is the most common cause of feline ureteral obstruction [4,5]. Owing to the narrow anatomical structure of the feline ureter and given that stones in the upper urinary tract are predominantly insoluble calcium-containing substances [2,6,7], treatment of feline ureteral obstruction is difficult. In addition, lithotripsy, which is utilized in human medicine, cannot be easily administered to cats because their ureters are narrow [7]. For this reason, various surgical approaches, such as ureterolithotomy, ureteral stenting, and subcutaneous ureteral bypass (SUB), have been attempted for treating feline ureteral obstruction [4,5,8]. However, these surgical approaches require anesthesia and have several disadvantages, such as high cost, infection, re-obstruction, and urine leakage [5,8]. Medical treatments, such as intravenous fluid therapy, osmotic diuresis, and ureteral muscle relaxation drugs, which cost less than surgeries, can be attempted to treat ureteral obstruction in cats [9]. However, a careful approach is required, as the obstruction may not improve or may become more severe during medical treatment. Clinical veterinary data from various trials will help determine the appropriate medical or surgical approach for the treatment of ureteral obstruction. However, compared with meta-analyses on expulsion therapies in humans, the available clinical veterinary data on the efficacy of drugs that relax the ureter, such as alpha-receptor antagonists, are limited [9,10]. A systematic review of medical therapy for facilitating the passage of ureteral calculi indicated that medical expulsive therapy using an alpha antagonist or a calcium channel blocker increases the possibility of the expulsion of small-sized (<5 mm) distal ureteral stones [10]. Considering the results of studies in humans and differences in ureter sizes in humans and cats, medical treatment can be effective in cats with stones smaller than 2 to 3 mm [7]. However, although tamsulosin has been used according to anecdotal evidence, reports on the results of using drugs in cats are insufficient [11,12]. Thus, this study aimed to investigate the factors affecting the outcome of tamsulosin, an alpha-receptor antagonist, for the treatment of feline ureterolithiasis. The results of administering tamsulosin in cats with obstructive ureterolithiasis with various conditions will help establish a treatment strategy for cats with similar diseases.

## 2. Materials and Methods

### 2.1. Study Design and Patient Selection

This study involved the retrospective analysis of the electronic medical records of client-owned cats with obstructive ureteral stones that received an alpha-adrenergic antagonist at Western Referral Animal Medical Center, Seoul, Korea, between January 2018 and March 2022. The cats in this study underwent blood analysis, urinalysis, radiography, and abdominal sonography performed using an Aplio 400 ultrasound machine (Canon Medical Systems Corporation, Tokyo, Japan). The imaging results were interpreted by experienced veterinarians specialized in imaging studies. Information obtained from medical records included signalment, location and size of the ureteral calculi, treatment methods, and stone passage status.

### 2.2. Treatment Protocol

The cats in this study received medical therapy as the initial treatment for ureteral obstruction. The medical treatment included restoring the intravascular volume using intravenous fluid therapy and ureteral muscle relaxation drugs. Tamsulosin (Hanmi Pharmaceutical Co., Ltd., Seoul, Korea) was administered to relax the ureteral muscles in doses ranging from 0.004 mg/kg to 0.006 mg/kg every 12 or 24 h [13]. Since the drug used was a 0.4 mg capsule used in human patients, the 0.4 mg dose was adjusted to the required amount and administered according to the weight of the cat.

Surgical intervention was not performed first owing to reasons such as financial issues, small-sized stones in the ureter, or refusal by the owner. Surgical interventions, such as SUB, were performed later for some cats if the location of the stone did not change even after medical treatment.

### 2.3. Evaluation of Treatment Response

Treatment response was analyzed based on changes in the locations of stones on ultrasound imaging. In some cats, stones were confirmed by radiographs, but most of them were smaller than 3 mm, so the change in the location of the stones was confirmed through ultrasound findings. If the renal pelvic dilatation due to the stone causing obstruction was resolved along with the movement of the stone on ultrasound, cases were classified as stone passage success group. In contrast, if there was no improvement in renal pelvis dilation due to occlusion and no displacement of stones, they were classified as the stone passage failure group. Observation period and intervals depended on the risk of occlusion due to stones. In the case of cats without clinical signs with unilateral partial occlusion, tamsulosin was prescribed as outpatient treatment, and the degree of renal pelvic dilatation according to obstruction and location of stones was evaluated at revisit. The revisit interval was determined by the attending veterinarian according to the degree of obstruction and serological azotemia in the cats.

### 2.4. Statistical Analysis

Univariable analysis based on Student’s t-test, Mann–Whitney U-test, and chi-squared test was used to determine the association between stone passing success and failure groups.

The multivariable logistic regression model was used to determine relationships between the passage of stones and baseline characteristics (sex, age, weight, diameter, and creatinine), excluding information on the location and side of stones. For statistical analysis, statistical software R was used for both methods. The resulting *p*-value less than 0.01, 0.05, and 0.1 will be considered statistically highly significant, statistically significant, and statistically provisionally significant, respectively. As such, a predictor with a smaller *p*-value is a baseline factor with higher statistical significance to differentiate the successes and failures of stone passage.

## 3. Results

### 3.1. Patient Characteristics

A total of 70 cats with confirmed ureterolithiasis who received tamsulosin were included in this study. Table 1 details the characteristics of the cats. The median age was 8.77 years (range 2–16 years), and most cats were male (55.7%). All cats investigated in this study were neutered. The median weight was 4.25 kg (range 1.9–10.3 kg). The breeds of cats were Korean Short Hair (*n* = 22), Siamese (*n* = 11), Persian (*n* = 9), Russian Blue (*n* = 7), Turkish Angora (*n* = 6), American Short Hair (*n* = 5), Scottish Fold (*n* = 4), British Short Hair (*n* = 1), Munchkin (*n* = 1), Ragdoll (*n* = 1), Chinchilla (*n* = 1), Abyssinian (*n* = 1), and Norwegian Forest (*n* = 1). In the evaluation of the location of ureterolithiasis, 26, 34, and 26 stones were in the upper, middle, and lower ureters, respectively. Bilateral ureteral stones were identified in 16 cats. Of these cats, 14 belonged to the stone passage failure group (Table 2).

### 3.2. Diagnostic Tests

Several tests were performed to determine the severity of obstructive ureterolithiasis.

Serum biochemistry and ultrasound were performed in 70/70 cats (100%). Azotemia (creatinine > 1.6 mg/dL) was present in 64/70 patients (six cats showed non-azotemic serological results due to partial obstruction of ureteral stones or compensatory response of the contralateral kidney, one cat from the stone passage success group and five from the failure group).

Urinalysis was performed in 54/70 cats (77.14%). Urine culture was performed in 34/70 cats (48.57%). Most culture tests were negative (*n* = 28), but six cats had positive culture test results. Bacteria identified were *Escherichia coli* (*n* = 2), *Enterococcus faecalis* (*n* = 1), *Candida tropicalis* (*n* = 2), and *Proteus mirabilis* (*n* = 1). Except for two cats in which *enterococcus* and *Candida tropicalis* were cultured, all four cases were cats of the stone passage success group.

### 3.3. Treatment Results

Depending on the location of the stone and the degree of obstruction, the clinical signs of cats varied from nonclinical signs to severe uremic crisis. In cases with severe obstruction, immediate surgical intervention was recommended as previously recommended [14,15]. However, owing to the owner’s cost burden and anesthesia risk, medical treatment using tamsulosin was first attempted.

Among the cats investigated in this study, the stone passage was confirmed in 22 cats, and no change in the position of stones was observed during follow-up in the remaining 48 cats. Of the 48 cats in the stone passage failure group, 19 underwent surgery (SUB: *n* = 16, ureteral stenting with stone removal: *n* = 1, nephrectomy: *n* = 1) to resolve ureteral obstruction.

The treatment response through the movement of stones was rechecked at short intervals during hospitalization in patients with severe clinical symptoms (46 cats, of which 17 were in the stone passage success group and 31 in the stone passage failure group). Patients with asymptomatic or non-serious clinical symptoms were managed through outpatient treatment; stone location and resulting renal pelvic dilation were monitored by ultrasound at each visit (24 cats, of which 7 were in the stone passage success group and 17 in the stone passage failure group). The revisit interval varied from 1 to 30 days depending on the degree of ureteral obstruction and serological azotemia in the cats.

The duration of tamsulosin administration and observation period depended on the success or failure of the stone passage and the condition of the patient (range 1–420 days). Interestingly, some cats (*n* = 3) were administered long-term tamsulosin because either renal pelvic dilation became severe, or obstruction recurred upon discontinuation of tamsulosin.

On univariate analysis (Table 2), the vertical diameters of stones, sex, and the location of stones were significantly associated with stone passage success. The median stone diameter measured using high-definition ultrasound were 1.09 ± 0.29 mm (range 0.55–1.65 mm) and 1.33 ± 0.49 mm (range 0.49–3.10 mm) for the stone passage success and failure groups, respectively (*p* = 0.0301, Table 2). The stone passage success group had a higher rate of stones in the lower ureter than the stone passage failure group (*p* = 0.0002, Table 2). The percentage of males in the stone passage success was higher than that in the stone passage failure group (*p* = 0.0140, Table 2). No significant difference was observed in the daily dose of tamsulosin between the stone passage success and failure groups (*p* = 0.3382).

On multivariate analysis (Table 3), important risk factors associated with the passage of stones were sex (odds ratio, 0.17; 95% CI, 0.04–0.59; *p* = 0.0008), vertical diameter of stone (odds ratio, 0.24; 95% CI, 0.05–0.90; *p* = 0.056), creatinine (odds ratio, 0.89; 95% CI, 0.78–0.99; *p* = 0.070). The results show (1) 83% higher odds of stone passage in males than in females; (2) 76% higher odds of stone passage as the stone diameter decreases by 1 mm; and (3) 11% higher odds of stone passage as the creatinine level decreases by 1 mg/dL.

In one of the cats in the stone passage failure group, there was no difference between the size of the stone extracted through surgery and the size observed on ultrasound images. The diameter of the stone as measured using ultrasound was 3.2 × 2.3 mm^2^.

**Figure 1:**
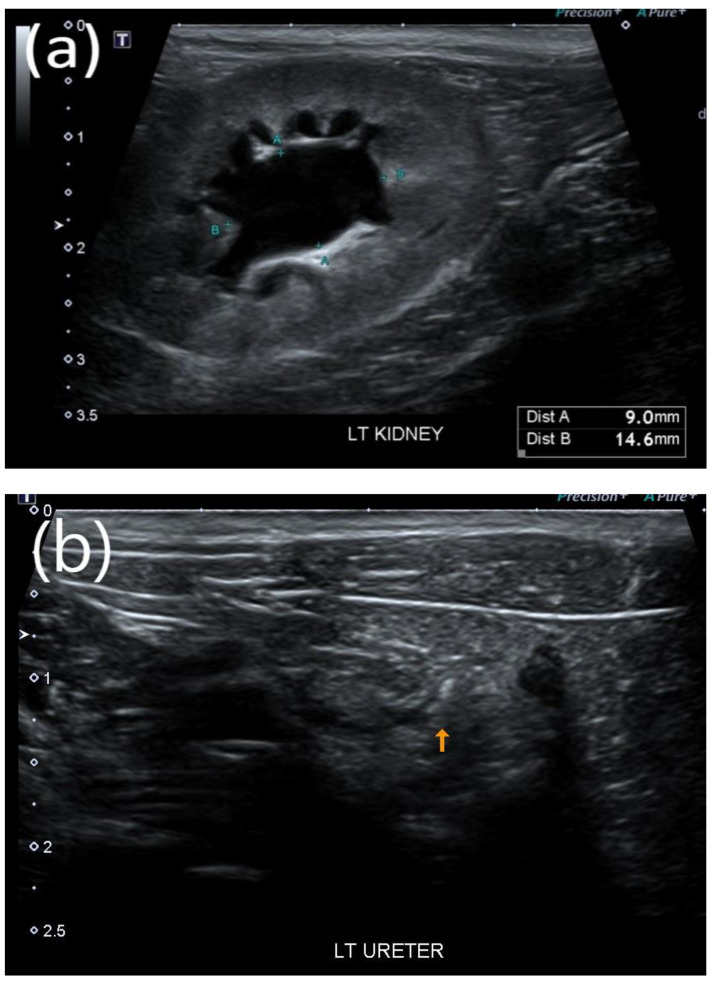

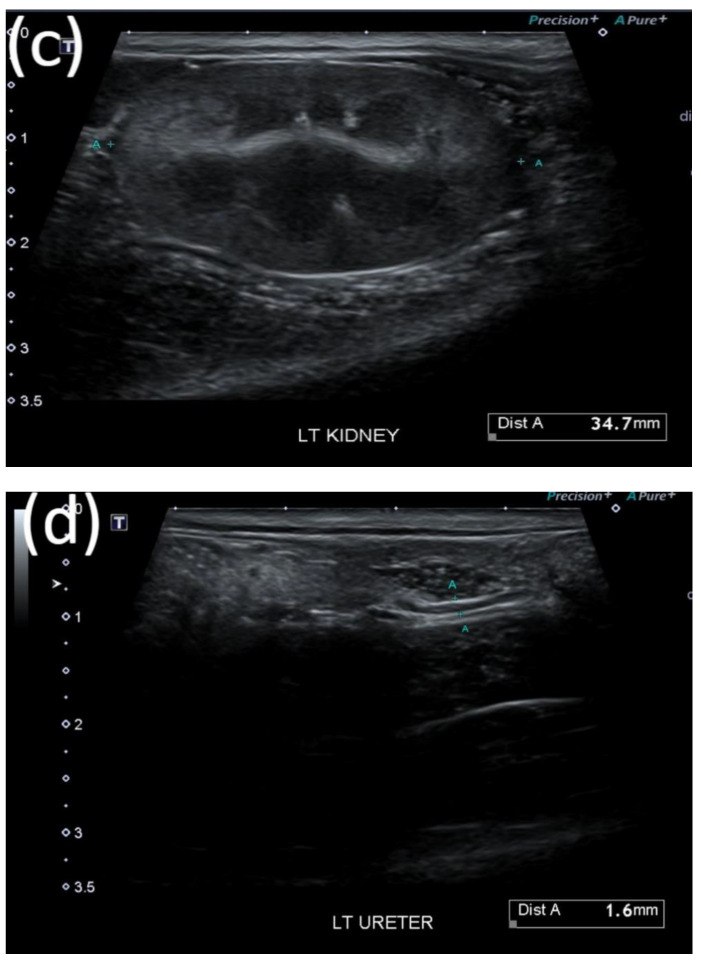
Representative ultrasound image of successful stone passage in a cat. (**a**) Dilated renal pelvis owing to obstruction and (**b**) high-contrast material presumed to be a stone in the distal part of the ureter. (**c**,**d**) After tamsulosin therapy, improved renal pelvic dilation and successful stone passage were confirmed through ultrasound imaging.

**Figure 2:**
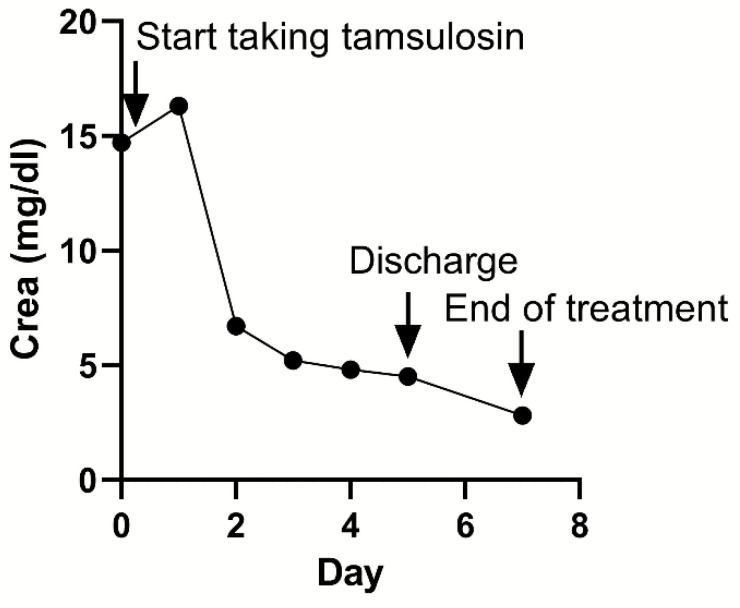
Improvement in serological azotemia with changes in the location of the stone observed on ultrasound.

After surgical removal, the stone, which contained calcium oxalate, was confirmed to have a diameter of approximately 3 × 2 × 1 mm^3^. For cats in the stone passage success group, changes in the locations of the stones and improvement in the extent of renal pelvis dilatation owing to obstruction were observed on ultrasound (Figure 1a–d). In one case where bilateral ureteral stones resolved simultaneously, treatment was successfully terminated with a dramatic decrease in creatinine (Figure 2).

### 3.4. Adverse Events

A known adverse event of tamsulosin is a sudden drop in blood pressure, which can cause dizziness or fainting [16,17]. However, no studies have been conducted to investigate the safety and adverse effects of tamsulosin in cats. For this reason, many cases were prescribed with a small initial dose referring to the dose cited in a drug book [13]. During the treatment period, serial monitoring was performed for possible adverse events such as a sudden drop in blood pressure after receiving tamsulosin. In addition to the sudden drop in blood pressure, adverse events known to occur when using tamsulosin in humans, such as vomiting and severe allergic reaction, were carefully monitored [17,18].

None of the cats experienced drug-related complications. No severe or serious adverse events occurred. Similarly, no adverse events occurred even when the maximum doses (0.006 mg/kg per 12 h) were administered.

## 4. Discussion

Since medical treatment was rarely effective, surgical procedures such as SUB or ureteral stenting have been recommended as a consensus treatment for feline obstructive ureteral stones [14]. Despite the consensus recommendations [14], there may be cases in which medical treatment should be attempted for various reasons, such as cost burden and risk of anesthesia during surgery. This study was conducted to evaluate the factors affecting the outcome of medical treatment in cats with obstructive ureterolithiasis treated with tamsulosin. Among the 70 cats with ureterolithiasis who underwent medical treatment, the displacement of stones was observed without surgery in 22 cats (31.43%). This percentage was higher than that reported in a previous study (8–17%) [15]. This relatively high percentage may be attributed to the following reasons: 1) the size of stones measured by ultrasound was limited to 2–3 mm, and 2) the use of tamsulosin was accompanied by relaxation of ureter muscle. The results showed that sex, baseline creatinine, and the location and diameter of stones were significantly associated with stone passage success. Negative factors with statistical significance for stone passage in this study were high baseline creatinine levels, female, proximal location of stones, and large diameter stones. The results suggest that tamsulosin may be used to treat ureteral obstruction in cats with small distal ureteral stones. To our knowledge, this is the first study on the results of tamsulosin for the treatment of obstructive ureterolithiasis in cats. Our results suggest that successful stone passage may be possible without surgical treatment in some cats with obstructive ureterolithiasis.

Several meta-analyses of expulsion therapies in humans have indicated that urolithiasis can be treated using medical treatments, such as fluid therapy, osmotic diuresis, and ureteral muscle relaxation drugs, depending on the size and location of the stone [10]. Moreover, it has been reported that ureterolithiasis in patients with small-sized stones less than 5 mm located in the distal ureter can be treated using non-surgical methods, such as the administration of alpha-antagonists [10,19]. In addition, studies have been conducted to evaluate the rate of spontaneous stone passage according to the size of the stones [20]. Owing to several studies on factors affecting the spontaneous passage of ureteral stones [21,22,23], a rational treatment can be selected according to the patient’s clinicopathological characteristics, including the location and size of the stones.

Extrapolation of the results of human research for minimally invasive management of uroliths of a specific diameter (≤2–3 mm) located in the distal ureter of felines has been suggested [7]. However, this extrapolation is only an option suggested based on the difference in the anatomical size of the ureter, and studies on the results of various therapeutic trials in cats are lacking. Owing to the lack of relevant research results, no clear treatment guidelines have been presented compared to human medicine. In addition, there have been no reports on the efficacy of alpha-antagonists for the treatment of feline ureterolithiasis.

Tamsulosin is an alpha-antagonist that relaxes the smooth muscles of the prostate, bladder, and ureter [9,17]. This drug is widely used for alleviating lower urinary tract symptoms and improving maximal urine flow in human patients with benign prostatic hyperplasia. Dizziness and abnormal ejaculation are the most common adverse events of tamsulosin treatment [17]. However, there have been no reports of its use in cats with urinary tract diseases. Although the recommended dose in cats has been reported in the veterinary drug handbook [13], information on drug half-life in feline species and its adverse events is limited. Owing to this lack of information, the daily dose of tamsulosin determined by each veterinarian in this study varied from 0.004 mg/kg to 0.012 mg/kg. As a result of using tamsulosin on 70 cats in this study, no adverse events caused by tamsulosin, such as hypotension and lethargy, were observed. Based on the safety results in this study and a systemic review of medical therapy to facilitate the passage of ureteral calculi in human medicine [10], we suggest that medical treatment with the use of tamsulosin can be selected instead of surgical treatment for small-sized distal ureteral stone in cats. However, to prove the efficacy of tamsulosin for feline ureterolithiasis, additional prospective studies including a control group and studies on the dose of drugs that cause significant ureter dilation in feline species should be conducted.

In the present study, we analyzed the outcomes of the treatment of ureterolithiasis in cats using tamsulosin. The results suggested that it is possible to induce stone passage and improve azotemia in some cats without surgical intervention. There were significant differences in the sizes of the stones measured using ultrasound and the locations of the stones between the groups. With reference to our results, based on the vertical diameter and location of the stone measured through ultrasound, the possibility of stone passage through medical treatment can be predicted.

The results of the present study showed that more male cats showed stone passage success than female cats. This finding may be attributed to sex-related differences in the anatomical diameter and structure of the ureter or a difference in response to drugs. However, additional research is needed to confirm this theory.

Although the inclusion criterion for the present study was not limited to the presence of distal ureter stones on computed tomography scans as in human studies [24,25], the findings of this study suggest that medical treatment with tamsulosin may be a safe option for feline patients with small distal ureter stones. If the cases included are only with stones in the distal ureter, a higher treatment success rate is expected. The results of this study will help suggest guidelines for cases in which medical treatment is indicated rather than surgical procedures.

This study has some limitations. First, the stone diameter was measured using ultrasound, which has several variables that may affect accuracy. However, this disadvantage was minimized because the stone size was measured by veterinarians with extensive experience in high-resolution ultrasound imaging. In one case of stone passage failure, the size of the stone measured after surgical removal did not differ from that measured using ultrasound. The second limitation was that, as mentioned previously, a control group was not included in this study. If there was a group in which tamsulosin was not used, the efficacy of tamsulosin could be demonstrated. It was difficult to prove the efficacy of tamsulosin. Additional prospective studies on cats with similar conditions treated using tamsulosin are required. Another limitation is the relatively small sample size, which may have influenced the greater number of male cats with distal ureteroliths that were able to pass the lith. Further studies with a large number of samples are warranted to accurately analyze the effects according to the sex of the cats. Finally, the treatment protocol was not unified owing to the scarcity of related studies on cats. Tamsulosin was administered in doses ranging from 0.004 mg/kg to 0.006 mg/kg every 12 or 24 h based on the information in previous veterinary studies [13]. In addition, the duration of tamsulosin administration varied from 1 day to 420 days depending on the degree of obstruction of the ureter. Although no major adverse events were observed in this study, additional research on various doses and regimens is needed to verify the optimal dose for treatment in similar cases.

Despite the above limitations, it is believed that tamsulosin will have a positive effect in resolving acute and chronic urinary tract obstruction by relaxing the ureter muscle. This can be known based on the higher probability of passing stones in this study than that in the previous reports and the cases of long-term prescription as the recurrent renal pelvic dilation improved upon re-administration of tamsulosin in this study.

## 5. Conclusions

In this study, we evaluated the factors affecting the outcome of tamsulosin treatment in cats with obstructive ureterolithiasis and found that tamsulosin was safe and effective at the dosages used for the cats. The results of this study suggest that tamsulosin can be considered for the treatment of ureterolithiasis in cats with small distal ureteral stones.

In addition, this study serves as an important reference for determining whether medical treatment of feline ureteral obstruction using tamsulosin can be attempted. The results of this study will help predict the possibility of stone passage during the medical treatment of cats with obstructive ureterolithiasis.

## Figures and Tables

**Table 1:** Cat demographics.

Characteristics	Cat Demographics (*n* = 70)
Age, median (range)	8.77 years (2–16)
Sex	
Castrated males	39 (55.7%)
Spayed females	31 (44.3%)
Weight, median (range)	4.25 kg (1.9–10.3)
Breeds	
Korean Short Hair	22
Siamese	11
Persian	9
Russian Blue	7
Turkish Angora	6
American Short Hair	5
Scottish Fold	4
British Short Hair	1
Munchkin	1
Ragdoll	1
Chinchilla	1
Abyssinian	1
Norwegian Forest	1
Locations of ureteral stones	
Upper	26
Middle	34
Lower	26

**Table 2:** Comparison of baseline characteristics for location and side of the stone.

Parameter	Passage+	Passage−	*p*-Value
Age (years)	7.82 ± 3.32	9.21 ± 3.64	0.1321 *
Vertical diameter of the stone (mm)	1.09 ± 0.29	1.34 ± 0.50	0.0301 ^†^
Location, *n* (%)	Upper 3 (12.5%) Mid 6 (25.0%) Distal 15 (62.5%)	Upper 23 (37.1%) Mid 28 (45.2%) Distal 11 (17.7%)	0.0002 ^‡^
Side, *n* (%)	Left 12 (54.5%) Right 8 (36.4%) Bilateral 2 (9.1%)	Left 21 (43.8%) Right 13 (27.1%) Bilateral 14 (29.2%)	0.1767 ^‡^
Sex, *n* (%)	Male: 17 (77.3%) Female: 5 (22.7%)	Male: 22 (45.8%) Female: 26 (54.2%)	0.0140 ^‡^
Body weight (kg)	4.16 ± 1.77	4.12 ± 1.41	0.5999 ^†^
Baseline serum creatinine levels	5.09 ± 4.14	7.34 ± 6.44	0.1338 ^†^

* Student’s *t*-test. ^†^ Mann–Whitney U-test. ^‡^ Chi-square test.

**Table 3:** Multivariate predictors of passage of stones.

Predictors	Odds Ratio	95% CI	*p*-Value
Age	0.90	0.75–1.06	0.2
Female sex	0.17	0.04–0.59	0.0008
Weight	0.78	0.50–1.16	0.2
Diameter	0.24	0.05–0.90	0.056
Baseline serum creatinine levels	0.89	0.78–0.99	0.068

CI: confidence interval.

## Data Availability

The data not presented in the manuscript are available for consultation after a reasonable request to the corresponding authors.
